# Antimicrobial peptides in livestock: a review with a one health approach

**DOI:** 10.3389/fcimb.2024.1339285

**Published:** 2024-04-24

**Authors:** Oscar Robles Ramirez, Gabriel Osuna, Fabien Plisson, Carolina Barrientos-Salcedo

**Affiliations:** ^1^ Doctorate in Agricultural Sciences, Facultad de Medicina Veterinaria y Zootecnia (FMVZ) Universidad Veracruzana, Veracruz, Mexico; ^2^ Irapuato Unit, Department of Biotechnology and Biochemistry, Center for Research and Advanced Studies of the National Polytechnic Institute (CINVESTAV-IPN), Irapuato, Mexico; ^3^ Medicinal Chemistry and Chemogenomics Laboratory, Facultad de Bioanálisis, Universidad Veracruzana, Veracruz, Mexico

**Keywords:** antimicrobial peptides, veterinary medicine, mastitis, One Health, bacterial resistance

## Abstract

Antimicrobial peptides (AMPs), often referred to as nature’s antibiotics, are ubiquitous in living organisms, spanning from bacteria to humans. Their potency, versatility, and unique mechanisms of action have garnered significant research attention. Unlike conventional antibiotics, peptides are biodegradable, adding to their appeal as potential candidates to address bacterial resistance in livestock farming—a challenge that has been under scrutiny for decades. This issue is complex and multifactorial, influenced by a variety of components. The World Health Organization (WHO) has proposed a comprehensive approach known as One Health, emphasizing the interconnectedness of human-animal-environment relationships in tackling such challenges. This review explores the application of AMPs in livestock farming and how they can mitigate the impact of this practice within the One Health framework.

## Introduction

**Figure 1:**
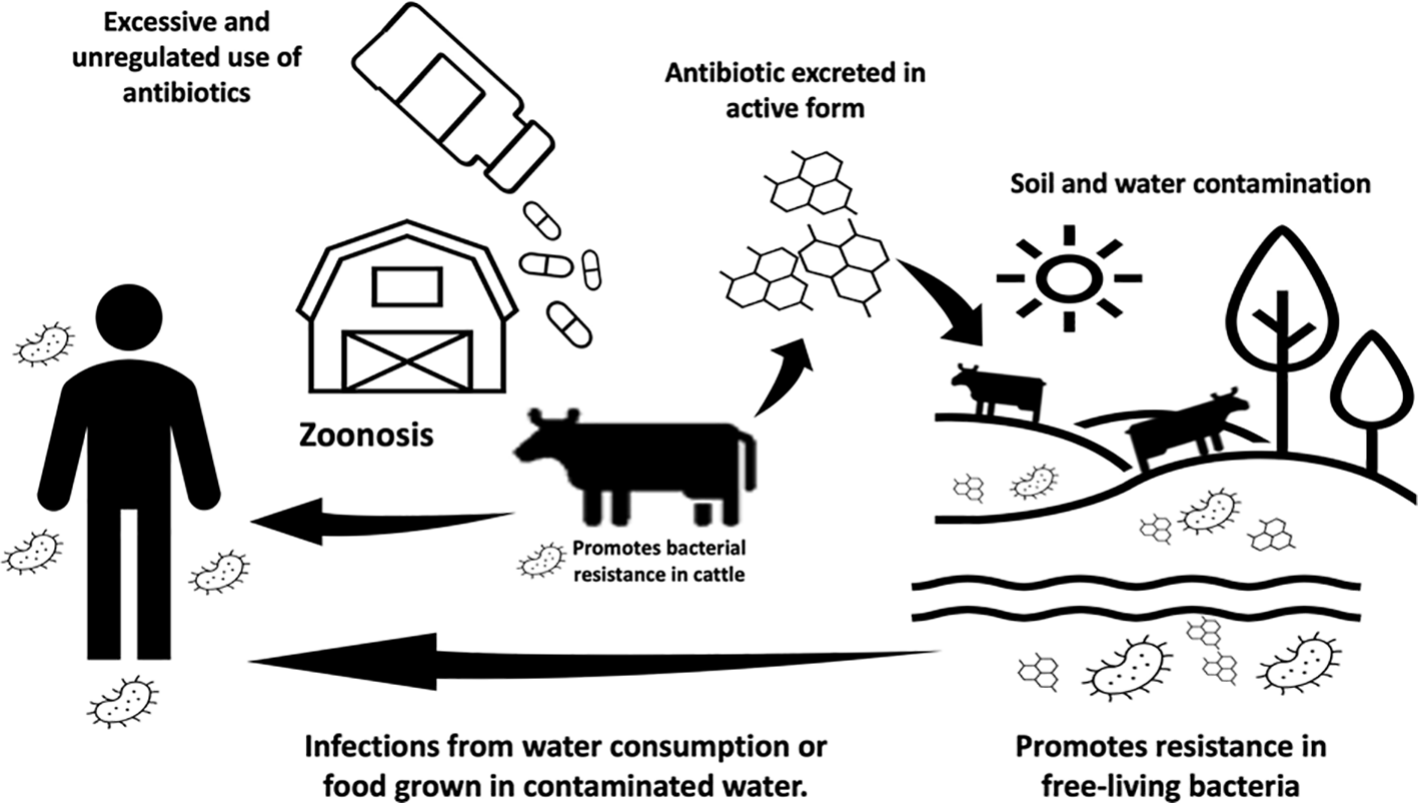
Schematic pathway and ramifications of antibiotic contamination in animals and the environment.

Conventional antibiotics have been extensively used for metaphylaxis and as growth promoters in various forms of livestock farming. The correlation between administering antibiotics at sub-therapeutic doses and enhanced animal weight gain is the driving force behind this practice (Dodds, 2017). These sub-therapeutic doses create a favorable environment for beneficial bacteria while impacting intestinal mucosa and motility, *i.e.*, reducing undesirable pathogens and nutrient wastage (Bacanlı and Başaran, 2019) and improving overall animal health (Palma et al., 2020). However, the use and abuse of antibiotics exert selective pressure on microorganisms, contributing to the surges of antimicrobial resistance (AMR) within intensive animal food production systems. Metaphylaxis, in particular, extends this selective pressure to entire animal groups when an infection is identified in one individual (Economou and Gousia, 2015). In both cases, sensitive microorganisms in infected animals or asymptomatic carriers among healthy animals develop resistance (**Figure 1**) (Mellor et al., 2019).

The rise of AMR has direct and indirect consequences on our public health and global economy. The direct consequences of AMR include impacts on food security and animal health, with infected animals facing slaughter or mutilation in the absence of effective treatment. Indirect consequences include the associated costs of treating and quarantining infected animals and the public health risks of drug-resistant pathogens with zoonotic potential. The World Health Organization (WHO) has proposed a unifying strategy known as One Health to prevent emerging zoonoses such as H1N1 flu and Hendra virus (Becker et al., 2022). The organization suggests shifting the current paradigm in our global food systems by recognizing the interdependence between the health of humans, domestic and wild animals, and the wider environment. This approach relies on the collective participation of communal, federal, and national entities to ensure the surveillance, regulation, and policy over antibiotic use (**Figure 1**) (Schmidt et al., 2017; Algammal et al., 2020; Bolte et al., 2020; Maity and Ambatipudi, 2021; Molineri et al., 2021; Velazquez-Meza et al., 2022). By working together, government and industries can identify emerging threats and develop more effective therapies.

Most organisms produce antimicrobial peptides (AMPs) as defense mechanisms in response to pathogenic infections. AMPs have gradually emerged as promising alternatives within the One Health approach in addressing the global challenge of AMR (Magana et al., 2020). First, the peptides exhibit broad-spectrum microbicidal activities against various pathogens, including bacteria, fungi, parasites, and viruses (Mookherjee et al., 2020). This makes them invaluable in both human and veterinary medicine. Second, AMPs do not induce bacterial resistance mechanisms due to their non- specific mechanisms of action on the bacterial membranes (Anaya-López et al., 2013; Assoni et al., 2020). This property is crucial in the fight against AMR, aligning with the One Health approach by offering a sustainable solution across human, animal, and environmental health. Finally, AMPs occur in Nature and are often biodegradable, posing fewer ecological risks than conventional antibiotics, which can persist and promote resistance development in microbial communities. By reducing environmental contamination, AMPs support the ecological aspect of the One Health approach. In summary, AMPs represent sustainable, broad-spectrum, and environmental- friendly alternatives to conventional antibiotics, directly supporting the goals of the One Health approach. Here, we describe the promising uses of antimicrobial peptides in veterinary medicine.

## Environment and antibiotic bioaccumulation

Antibiotics, much like plastic and fossil fuels, are among the significant advancements of the 20th century that were developed and utilized for their immediate value without a comprehensive understanding of their long-term environmental and biological impacts. Consequently, the presence of antibiotics in various natural environments, including lakes, rivers, and fields fertilized with biosolids, is well-documented in current scientific literature (Storteboom et al., 2010; Booth et al., 2020). The persistence of antibiotics in the environment (**Figure 1**) is a concern because a substantial proportion of antibiotics is not entirely metabolized and is excreted while retaining their activity (Sukul et al., 2009). These residual antibiotics persist in natural settings, interacting with various bacteria. This includes organisms such as Escherichia coli, which can originate from wastewater discharges, and free-living bacteria like *Vibrio cholerae* (Jang et al., 2017; De, 2021). In these interactions, residual antibiotics can trigger various biological mechanisms that promote bacterial resistance. Among these mechanisms are selective pressure and the exchange of mobile genetic elements. Thus, the environment plays a crucial role in disseminating antimicrobial resistance (AMR) (Williams et al., 2016).

## Zoonoses and the spread of antimicrobial resistance

Zoonoses are transmissible diseases from animals to humans, such as the well-documented examples of anthrax caused by *Bacillus anthracis*, bovine tuberculosis by *Mycobacterium tuberculosis*, brucellosis by *Brucella abortus*, and hemorrhagic colitis by *Escherichia coli* (Rahman et al., 2020). Beyond bacteria, zoonotic pathogens also include viruses (*e.g.*, Hendra virus, influenza virus A) (Leifels et al., 2022) and parasites (e.g., *Trypanosoma cruzi* and *Toxoplasma gondii*) (Weiss, 2008). These pathogens can reach humans through direct contact with food, water, or the environment (**Figure 1**). They represent a global health threat due to our close relationships with animals in agriculture, as companions, and in the natural environment. Zoonoses disrupt our current food production systems, leading to the spread of foodborne outbreaks (Abebe et al., 2020; Preena et al., 2020). Zoonoses have also become the sources of AMR with pathogens like extended-spectrum beta- lactamase (ESBL)-producing *E. coli* and methicillin-resistant *Staphylococcus aureus*), becoming notably resistant to our antibiotics. Comprehensive reviews have documented the connections between zoonotic diseases and the spread of antimicrobial resistance (Srivastava and Purohit, 2020; Olaru et al., 2023). The indirect transmission of zoonoses involves vector insects or pets acting as a bridge between production areas and households (Bolte et al., 2020). The need to discover antibacterial molecules that are environmentally non-persistent has become a top priority. Antimicrobial peptides (AMPs) emerge as potential alternatives to contemporary antibiotics. To understand the progress in this domain, we listed peptides tested in livestock production (**Table 1**), considering associated bacteria for different livestock types and emphasizing achieved outcomes.

**Table 1:** List of AMPs used as antibiotics, supplements, and food preservatives in animal products.

	Source	Peptide	Target	Application	Reference
Swine	*B. subtilis*	NRWCFAGDD	*H. parasuis*	Antibiotic	(Teixeira et al., 2013)
	Milk	Lactoferricin	*E. coli*	Suplement	(Tang et al., 2011)
	*B. subtilis*	Surfactins	*B. hydrodysenteriae*	Antibiotic	(Horng et al., 2019)
	*B. licheniformis*	Surfactins	*C. perfringens*	Antibiotic	(Horng et al., 2019)
Goat	Pigs/flies	PBD–mI/LUC–n	*Selenomonas*, *Succinivibrio*, *Treponema, Polyplastron, Entodinium, Isotricha*	Suplement	(Liu et al., 2017)
	*B. thuringiensis*	Bacteriocins	*E. durans*, *B. spp.*, *P. spp.*, *P. brenneri.*	Antibiotic	(Gutiérrez-Chávez et al., 2016)
	Silk moth	Cecropin B	*S. aureus*	Antibiotic	(Luo et al., 2013)
Bovine	*P. nigrella*	Plectasin	*S. aureus*	Antibiotic	(Li et al., 2017)
	Bbpi	Bbpi90–99,148-161	*S. aureus, E. coli, P. aeruginosa*	Antibiotic	(Chockalingam et al., 2007)
	Lactoferrin	Lactoferricin	*E. coli, S. aureus, S. zopfii, yeasts*	Antibiotic	(Bruni et al., 2016)
	*S. aureus* *S. Epidermidis*	A53/a70	*S. aureus* y *S. agalactiae*	Antibiotic	(Varella Coelho et al., 2007)
	*B. Thuringiensis*	Morricin 269 Kurstacin 287	*S. aureus*	Antibiotic	(Barboza-Corona et al., 2009)
	*Streptococcus equinus*	Bovicin HC5	*S. aureus*, *S. agalactiae*, *S. bovis*, *S. uberis*	Food preservative	(Godoy-Santos et al., 2019)
	*L. lactis*	Nisin	*Staphylococcus spp*	Antibiotic	(Castelani et al., 2019)
	Cows	Tap	*S. aureus, E. coli*	Antibiotic	(Sharma et al., 2017)
Poultry	*B. subtilis*	Sublancina	*C. perfringens*	Antibiotic	(Wang et al., 2015)
	*Epinephelus lanceolatus*	Piscidina	*S. aureus*, *E. coli*, *P. aeruginosa*	Suplement	(Tai et al., 2020)

## Swine livestock

In swine, one of the most important bacteria in veterinary clinics is *Haemophilus parasuis*, which causes Glässer’s disease. Teixeira and colleagues, in 2013, isolated a peptide from the culture supernatant of *Bacillus subtilis* subsp. *spizezinii*. Characterization of the peptide revealed antimicrobial activity against *H. parasuis* (Teixeira et al., 2013). On the other hand, using peptides as dietary additives for pigs has proven beneficial, as demonstrated by Tang and colleagues in 2011. They analyzed the effect of dietary supplementation with 100 mg/kg of lactoferricin peptide in a model of gastrointestinal infections by *E. coli* in 21-day-old pigs.

The study yielded positive results for the animals, with one of the main effects being the alteration of the gastrointestinal microbiome. This led to beneficial consequences such as improved nutrient retention and intestinal morphology, reduced incidences of gastrointestinal diseases like diarrhea, and promoted animal growth. This treatment reduced the concentration of *E. coli* and increased the presence of commensal bacteria such as *Lactobacillus* and *Bifidobacterium*. Additionally, it counteracted the effects of E. coli on intestinal architecture by promoting the height of intestinal villi in the jejunum and ileum compared to the group without the peptide. Furthermore, lower concentrations of proinflammatory cytokines such as TNF-alpha, IL-1 beta, and IL-6 were found, along with higher concentrations of growth hormone in the treated pigs (Tang et al., 2011). Another group of peptides evaluated for swine pathogens were surfactins from *B. subtilis* and *Bacillus licheniformis* against intestinal pathogens *Brachyspira hyodysenteriae* and *Clostridium perfringens*, which cause swine dysentery and necrotic enteritis. In this group of peptides, both the dose-response relationship and treatment effectiveness were compared between them. The results showed that *B. subtilis* surfactin is more effective against *B. hydrodysenteriae*, while *B. licheniformis* surfactin is more effective against *C. perfringens*. This suggests that peptide isoforms between species may have selective effectiveness against specific pathogens (Horng et al., 2019).

## Goat livestock

Among the peptides studied in goats, researchers have explored recombinant porcine defensin PBD-mI and the peptide isolated from flies, LUC-n. The supplementation of these peptides resulted in a notable alteration of the rumen microbiome, as demonstrated by the analysis of 16S bacterial genes and 18S rRNA genes of ciliated protozoa. Post-treatment analysis revealed an increase in beneficial genera, such as *Fibrobacter*, *Anaerovibrio*, *Succiniclasticum*, and *Ophrysocolex*, while pathogenic genera, like *Selenomonas*, *Succinivibrio*, *Treponema*, *Polyplastron*, *Entodinium*, and *Isotricha* decreased. Additionally, there were changes in enzymatic activity, including xylanase, pectinase, and lipase (Liu et al., 2017). These peptides underwent further evaluation using a different experimental design. Goats were divided into three groups: a control group, one supplemented with 2 grams per day of a combination of AMPs, and another with 3 grams per day. Ren and colleagues conducted this study in 2019 with a distinct experimental setup but arrived at results similar to the previous study: changes in digestion translated into an increase in the mass of treated animals compared to the control group. It is noteworthy that the group administered with 2 grams per day of the peptide combination showed greater mass gain than those given 3 grams, leading to the conclusion that the administered peptide quantity does not linearly affect the increase in body mass of animals, at least in goats. Therefore, an appropriate dosage is more relevant than a high amount administered (Ren et al., 2019). Bacteriocins from *Bacillus thuringiensis* were also evaluated in clinical isolates of goat mastitis. Various pathogenic species, such as *Enterococcus durans*, *Brevibacillus* spp., *Enterobacter* sp., *Escherichia vulneris*, *Pantoea* spp., *Pseudomonas brenneri*, and encapsulated yeast *Cryptococcus neoformans*, as well as several *Staphylococcus* species, were identified. The microbiocidal activity was observed in 67% of these isolated bacteriocins. However, species like *Staphylococcus epidermidis*, *Enterobacter* sp., *E. vulneris*, and *C. neoformans* proved resistant to all bacteriocins (Gutiérrez-Chávez et al., 2016). Finally, a novel perspective on mastitis treatment in goats involves the generation of peptides directly in milk. This is achieved by transfecting animals with plasmid vectors containing the peptide sequence. For instances, cecropin B, an AMP from the giant silk moth, was transfected into goat mammary glands, resulting in inhibitory effects against *S. aureus* (Luo et al., 2013).

## Bovine livestock

Cattle suffer from various zoonotic diseases, including tuberculosis (e.g., *Mycobacterium* bovina) and mastitis (e.g., *Corynebacterium diphtheriae*, *Staphylococcus aureus*), leading severe loss in animal production. Several peptides have been particularly evaluated to treat *S. aureus*-induced bovine mastitis and ulcers on teats, as the pathogen can reside intracellularly within mammary gland epithelial cells (Alva-Murillo et al., 2017). For example, the peptides NZ2114 and MP1102, derived from plectasin, an amphipathic peptide isolated from the fungus *Pseudoplectania nigrella*, were evaluated in murine models of *S. aureus*-induced mastitis and sterile milk cultures. Of note, milk components (among other pathophysiological factors) might significantly affect peptides (Schmelcher et al., 2015). As a result, the peptides were also tested on bovine mammary epithelial cells infected with this bacterium. Both peptides exhibited activity against *S. aureus* in sterile milk cultures, indicating that their effectiveness is not compromised in milk. They demonstrated intracellular activity against *S. aureus* without any cytotoxic effects at concentrations of up to 100 μg/mL. These peptides were also effective in experimental mastitis treatment (Li et al., 2017). Bacteriocins are another group of antimicrobial peptides that were evaluated against *S. aureus*-induced bovine mastitis. As such example, in 2009, Barbosa and colleagues determined that S. aureus AMR isolates obtained from milk of cows diagnosed with mastitis were sensitive to five bacteriocins from *B. thuringiensis*, with morricin 269 and kurstacin 287 exhibiting the greatest activity (Barboza-Corona et al., 2009). Likewise, bovicin HC5, a bacteriocin obtained from *Streptococcus equinus* HC5 (found in the horse gastrointestinal tract), possesses valuable characteristics for food preservation. It is a thermo-stable peptide with a mechanism of action described as lipid II-dependent, soluble at neutral pH, and effective even in acidic pH. This peptide inhibited the growth of mastitis-causing bacteria such as *S. aureus*, *S. agalactiae*, *Streptococcus bovis*, and *Streptococcus uberis* at concentrations ranging from 40 to 2560 arbitrary units (u.a.)/mL (Godoy-Santos et al., 2019). Finally, in 2021, Sharma and colleagues proposed the directed expression of the tracheal antimicrobial peptide (Tap) to treat *S. aureus*-associated mastitis in mice. They observed significant antibacterial effects in both *in vitro* and *in vivo* experiments by introducing a vector with the TAP peptide into mice infected with S. aureus associated with bovines (Sharma et al., 2021). Similarly, in 2017, the expression of a peptide derived from lactoferricin was carried out using the PiggyBac plasmid in bovine mammary epithelial cells, resulting in successful protection against *S. aureus* and *E. coli* (Sharma et al., 2017). The aforementioned peptides are all derived from external sources. Some peptides originally from cattle or their milk have also been studied for their antimicrobial properties. In 2007, Chockalingam and co-workers synthesized the hybrid peptide named bBPI90–99,148-161, where bBPI stands for bovine bactericidal permeability-increasing protein. This hybrid peptide had an average inhibitory concentration of 16 μg/mL against *E. coli* and 128 μg/mL for *Pseudomonas aeruginosa*, but was ineffective against *Serratia marcescens*. In addition, the antimicrobial activity of the peptide decreased after being suspended in milk, while it remained stable when tested in serum. These results indicated the importance of assessing different fluids in which the peptides may be administered (Chockalingam et al., 2007). Cow milk also holds a rich source of proteins and potential AMPs. Examples include beta- lactoglobulin, alpha-lactalbumin, and lactoferrin (Mohanty et al., 2016). The latter is a glycoprotein with iron- binding properties that plays a significant role in the bovine immune system. This 692- residue protein has demonstrated antimicrobial, anti-inflammatory, and immunomodulatory effects (Woodman et al., 2018). Lactoferricin is a small peptide derived from lactoferrin, which has been effective in treating subclinical mastitis caused by *E. coli and Staphylococci* in cattle. It also exhibited *in vitro* activity against an alga called *Prototheca zopfii*, responsible for protothecal mastitis, and yeast strains causing fungal mastitis (Bruni et al., 2016).

## Poultry industry

Clostridium perfringens is one of the most significant pathogens in the poultry industry, being the primary etiological agent of necrotic enteritis, a disease that causes substantial economic losses for producers (Ben Lagha et al., 2017). Factors predisposing to infection and various strategies to control the effects of this pathogen have been extensively studied (Allaart et al., 2013). In 2015, Wang and colleagues reported sublancin, a bacteriocin produced by *B. subtilis*, for its potential antimicrobial effect against *C. perfringens* in chickens. Their results revealed that the peptide displayed similar antimicrobial activity to the commercial antibiotic lincomycin. Unlike the group administered with sublancin, the lincomycin-treated group also experienced a reduction in *Lactobacillus* colonies, a commensal bacterium in the chicken digestive system. Sublancin might not affect other bacterial species in the avian intestinal microbiomes, but there is evidence of potentially greater specificity between species compared to conventional antibiotic treatments (Wang et al., 2015). Alternatively, peptides have also been employed as dietary supplements to promote growth in poultry. One such example is piscidin, isolated from the fish *Epinephelus lanceolatus*, which was utilized as a dietary additive and compared with control groups. Its antimicrobial activity against strains of *S. aureus*, *E.coli*, *P. aeruginosa*, and various strains of *Riemerella antipestifer* was evaluated, with the first three and some *R. antipestifer* strains proving sensitive to the peptide (Tai et al., 2020). Piscidin also exhibited immunomodulatory properties, increasing the secretion of interferon-gamma, immunoglobulins G, and interleukin-10 compared to the control group. Finally, the peptide induced significant changes in bacterial communities, increasing the families *Enterococcaceae* and *Lactobacilliaceae* while decreasing *Enterobacteriaceae* and *Staphylococceaceae*.

## Antimicrobial peptides in livestock-relevant clinical strains

Antimicrobial peptides have been used against important clinical strains of the same genus and species. For example, bacteriocins isolated from *S. aureus* and *S. epidermidis* against other strains of *S. aureus* and *Streptococcus agalactiae* isolated from bovine mastitis. In 2007, Varella Coelho and co-workers identified bacteriocin A53 had a moderate effect against some clinical strains. However, when used in combination with A70, a bacteriocin with a similar effect alone, the synergistic effect resulted in increasing the inhibition percentages in *S. agalactiae* (i.e., from 67.6% to 91.9%) and in *S. aureus* (i.e., from 74.4% to 91.5%) (Varella Coelho et al., 2007). These results suggest that antimicrobial peptides from pathogenic bacteria might be helpful against similar pathogens. Another bacteriocin named nisin from *Lactococcus lactis* has been noted for having high antimicrobial activity against Gram-positive bacteria. In 2019, Castelani and co-workers evaluated its antimicrobial potential against *Staphylococcus* spp. isolated from cases of bovine mastitis. The peptide exhibited antibacterial effects against antibiotic-resistant strains. In combination with dioctadecyldimethylammonium bromide, a quaternary amine with broad antimicrobial activity, it enhanced the susceptibility of isolates to the bacteriocin, reducing the minimum bactericidal concentration from 50 to 3 µg/mL (Castelani et al., 2019). These two examples showed that synergistic effects should be considered in future studies.

## Conclusions

The significance of developing therapies in veterinary medicine aligns with the “One Health” concept, acknowledging the interdependence of health across ecosystems, including livestock, pets, wildlife, and plants. Embracing this holistic approach, the urgency to invest in novel peptide-based antimicrobials becomes apparent. These peptides exhibit dual mechanisms of action: direct effects on microorganisms and stimulation of the host’s immune response, enhancing their effectiveness against infections. A particularly challenging scenario is observed in bovine mastitis, where the need for peptides capable of remaining stable in the presence of milk components is crucial. Regulatory challenges and concerns about resistance to AMPs complicate their applications in livestock. Despite these challenges, the slower pace of bacterial adaptation to these peptides compared to conventional antibiotics places AMPs as novel therapeutic agents against infections, extending beyond the realms of veterinary clinics and mastitis. AMPs offer a potential solution to the scarcity of effective antibiotics against multidrug-resistant bacteria, emphasizing the need for responsible antibiotic use across all sectors. Efforts to manage antibiotic use, including exploring strategies like genetically engineered microbes for environmental clean-up, are imperative for global health and food security.

## Author contributions

OR: Investigation, Writing – original draft. GO: Writing – review & editing. FP: Validation, Writing – review & editing. CB-S: Writing – review & editing, Conceptualization, Funding acquisition, Supervision.
